# Insights into Metabolite Diagnostic Biomarkers for Myalgic Encephalomyelitis/Chronic Fatigue Syndrome

**DOI:** 10.3390/ijms22073423

**Published:** 2021-03-26

**Authors:** Emi Yamano, Yasuyoshi Watanabe, Yosky Kataoka

**Affiliations:** 1 Laboratory for Pathophysiological and Health Science, RIKEN Center for Biosystems Dynamics Research, 6-7-3 Minatojima-minamimachi, Chuo-ku, Kobe, Hyogo 650-0047, Japan; yywata@riken.jp; 2 Laboratory for Cellular Function Imaging, RIKEN Center for Biosystems Dynamics Research, 6-7-3 Minatojima-minamimachi, Chuo-ku, Kobe, Hyogo 650-0047, Japan; kataokay@riken.jp; 3 Multi-Modal Microstructure Analysis Unit, RIKEN—JEOL Collaboration Center, 6-7-3 Minatojima-minamimachi, Chuo-ku, Kobe, Hyogo 650-0047, Japan

**Keywords:** ME/CFS, etiology, biomarker, metabolome, objective diagnosis

## Abstract

Myalgic encephalomyelitis/chronic fatigue syndrome (ME/CFS) is a persistent and unexplained pathological state characterized by exertional and severely debilitating fatigue, with/without infectious or neuropsychiatric symptoms, and with a minimum duration of 6 consecutive months. Its pathogenesis is not fully understood. There are no firmly established diagnostic biomarkers or treatment, due to incomplete understanding of the etiology of ME/CFS and diagnostic uncertainty. Establishing a biomarker for the objective diagnosis is urgently needed to treat a lot of patients. Recently, research on ME/CFS using metabolome analysis methods has been increasing. Here, we overview recent findings concerning the metabolic features in patients with ME/CFS and the animal models which contribute to the development of diagnostic biomarkers for ME/CFS and its treatment. In addition, we discuss future perspectives of studies on ME/CFS.

## 1. Introduction

Fatigue is experienced by humans on a daily basis and is an alarm that signals the disturbance of homeostasis in the body, caused by either excessive physical and mental activities or illness [1]. In a narrow sense, “fatigue” is a state of diminished ability and efficiency of activity caused by mental and physical overload, whereas “fatigue sensation” is defined as a feeling of awareness about the existence of fatigue and is often accompanied by a sense of discomfort, desire for rest, and reduced motivation towards any activities [2]. Many people experience fatigue that lasts for longer than 6 months (chronic fatigue) and some feel that their ability to work has declined and that they are not able to work well [1]. Chronic fatigue is also one of the major social problems from the viewpoint of economic loss.

Myalgic encephalomyelitis/chronic fatigue syndrome (ME/CFS) is a clinically complex chronic condition characterized by exertional and debilitating fatigue with a wide spectrum of symptoms including pain, cognitive dysfunction, autonomic dysfunction, sleep disturbance, and neuroendocrine and immune symptoms [3,4]. To be diagnosed with ME/CFS, a patient will meet the criteria for post-exertional neuroimmune exhaustion and experience a symptom from neurological impairments, a symptom from immune/gastro-intestinal/genitourinary impairments, and a symptom from energy metabolism/transport impairments. Such symptoms in a patient, however, fluctuate in intensity and severity and are heterogeneous, ranging from mild to severe, even leaving some patients bed-bound [3]. Identifying the characteristic abnormal factors for this condition is difficult using general and conventional medical examination, and no treatment has been established yet [5]. There is an overall delay from the appearance of symptoms to diagnosis. In other words, it might take several months or several years for the mental and physical changes to appear and lead to the diagnosis of ME/CFS.

To date, the onset mechanisms of ME/CFS has been assumed to be via viral infections, immune dysfunction, hypothalamic-pituitary-adrenal system (HPA axis), dysfunction, impaired oxidative phosphorylation, and oxidative stress [6,7,8]. In addition, there have been discussions about the abnormal activity of enzymes in the tricarboxylic acid (TCA) cycle, with some reports indicating reduced production of adenosine-5′-triphosphate (ATP) in the mitochondria [9,10,11]. Although the precise etiology of ME/CFS remains unknown, recent advances and discoveries have begun shedding light on the enigma of this disease, including the following contributors: infectious, genetic, immune, cognitive, sleep, metabolic, and biochemical abnormalities [12].

Symptoms in an individual person may fluctuate both in intensity and severity and there is also great variability across individuals [12]. The diagnosis, therefore, depends on symptom-specific case criteria following the exclusion of any other explanatory diagnosis [3,4,13,14]. So far, various studies have been conducted with the aim to elucidate the molecular mechanisms underlying the pathology of fatigue and to develop objective diagnostic methods, treatments, and preventive measures for ME/CFS [15]. There has been a constant need to establish diagnostic biomarkers that reflect its pathological mechanism and enable its objective diagnosis [15].

In recent years, an increasing number of studies have elucidated the pathology of ME/CFS and have developed biomarkers for the same by using a metabolome analysis approach [16,17,18]. Metabolite profiling provides direct functional information regarding metabolic profiles and indirect functional information regarding the arrangement of profiles determined by small molecules, such as disease manifestations [19,20]. Alterations in metabolites may be associated with the pathogenesis of diseases not previously considered to be of metabolic origin, such as cancer and cognitive disorders [17]. Therefore, a comprehensive analysis of metabolites has been used to characterize the pathophysiology of various disease states and thus assist in drug discovery, disease diagnosis, and treatments [21]. Based on the above considerations, we believe that an elucidation of the etiology reflecting the pathophysiology of ME/CFS and development of biomarkers useful for objective diagnoses based on the pathology might be possible using metabolomic analysis.

This review aimed to provide an overview of the findings from previous metabolomic studies involving animal models of fatigue as well as patients with ME/CFS and also suggested some future perspectives of studies on ME/CFS.

## 2. Metabolomic Analysis in an Animal Model of Fatigue

Metabolic alterations in an animal model of complex fatigue were investigated in order to understand the pathophysiology of fatigue in multiple organs. Metabolome analysis enables the identification of metabolites or metabolic pathways involved in disease pathophysiology. The efficacy of this experimental approach was demonstrated by the discovery of biomarkers for type 2 diabetes [22], chronic kidney disease [23], and Parkinson’s disease [24].

### 2.1. Rat Model of Fatigue

Fatigue sensation in humans may be caused by multiple factors, such as physical and mental tasks, mental/environmental stress, and lack of sleep. In order to investigate the molecular mechanisms underlying such fatigue, an experimental animal model that reproduces continuous stress load and sleep deprivation would be relevant. Therefore, Tanaka et al. reared rats in cages filled with water up to a depth of 1.5 to 2.2 cm for 3 to 7 days to create an animal model of fatigue [25]. Rats did not get enough rest and suffered from generalized fatigue as a result of continuous deprivation of rapid eye movement (REM) sleep, which is known to relieve muscle tension. In a forced swim test conducted on this animal model as a quantitative indicator of the degree of fatigue, the swimming time was found to be shortened significantly after fatigue loading [25]. In addition, abnormalities in the metabolism of brain monoamines, such as serotonin, and abnormalities in the neuro-immune-endocrine system, including overexpression and over-secretion of α-melanocyte stimulating hormone (α-MSH) from the intermediate lobe of the pituitary gland, were confirmed [26]. Furthermore, the content of 20 proteinogenic amino acids in the liver, muscle, brain, and plasma was found to be either increased or decreased [27]. Kume et al. carried out metabolomic analyses using the plasma from an animal model of fatigue, prepared by rearing rats in water-filled cages for 5 days, to investigate changes in metabolites that are specific to fatigue [28]. Eight-week-old male SD rats were reared in an environment with 12 h daylight, a room temperature of 23 °C and a humidity of 50%. They were then randomly allocated into a control group (non-fatigue load group), fatigue load group, and a dietary restriction group. Control group rats were reared in a normal cage without fatigue loading for the same number of days as the other rats. Rats in the dietary restriction group were reared in a normal cage for the same number of days as other animals while being given half of the normal quantity of food for intake (10 g per day of feed) and exhibiting the same level of weight loss as the animals in the fatigue load group, in order to distinguish the metabolic changes associated with reduced food intake. Metabolites were measured by capillary electrophoresis, time-of-flight mass spectrometry (CE-TOF-MS) and liquid chromatography-mass spectrometry (LC-MS) using plasma samples from rats in these 3 groups, and total nitrogen oxide (NOx) was also measured in the samples. In addition, the ATP levels in each tissue, such as the liver and muscles, were also checked [28].

In an analysis by both cation-mode and anion-mode methods of CE-MS, we identified 48 types of metabolites and confirmed an increase in lactate, succinate, and branched-chain amino acids (valine, leucine, isoleucine) in the fatigue load group. In terms of the urea cycle and proline metabolism, citrulline and hydroxyproline decreased in the fatigue load group than other experimental groups. Moreover, the measurement of citrate, *cis*-aconitate, and isocitrate levels in the first half of the TCA cycle by LC-MS under conditions of higher sensitivity showed that the citrate level tended to decrease, whereas the *cis*-aconitate and isocitrate levels decreased significantly in the fatigue load group compared with levels in the control group [28].

When the relationship between energy production and oxidative stress in tissues was investigated, ATP levels in the liver and muscle were significantly lower in the fatigue load group than in the control and dietary restriction groups. On the other hand, there was no change in ATP levels in the cerebral cortex and erythrocytes. In addition, the amount of NOx in plasma, which is an indicator of oxidative stress, was significantly increased in the fatigue load group than in the control group and the dietary restriction group. Based on the above results, changes by fatigue were inferred to involve increased systemic oxidative stress and reduced mitochondrial function [28].

### 2.2. Rat model of Excess Fatigue

Metabolic changes in excess-fatigue rats have also been reported [29]. The excess-fatigue model was established as follows: male Wistar rats weighing 180–200 g were raised with the basic diet (corresponding to 5% of body weight of the full-grown rats) and made to swim in a self-manufactured swimming cylinder, in which the water temperature was 26 ± 1 ℃. The rats swam twice every day with an interval of 10 min [29]. Each rat was hung with a clog (its weight was 5% of the rat’s body weight) on its tail while they swam. The clog did not stop the rat from swimming until they were exhausted, as evidenced by their motion being disordered and their heads being inside the water, and not outsidefor 10 s. Rats were made to swim for 14 consecutive days to develop the excess-fatigue model. Compared to the control rats, the excess-fatigue rats showed lassitude, with closed eyes and less activity, showing loss of body weight, luster-less hair, and pale tail. Their climbing time was significantly reduced in contrast to that of the control rats. The exhaustive swimming time on day 14 was also decreased markedly compared to that on the first day [29]. Using ultra-fast liquid chromatography coupled with ion trap-time-of-flight mass spectrometry (UFLC/MS-IT-TOF), plasma samples were analyzed. Spermine, propionylcarnitine, butyrylcarnitine, and phenylalanine were down-regulated significantly in the excess-fatigue rats [29]. Carnitine is an important carrier amino acid that transfers acyl-residue into the mitochondrial matrix and participates in fatty acid β-oxidation (TCA cycle, ATP production, and energy metabolism) [30]. In an open-label study involving 30 patients, acetyl-L-carnitine supplements were reported to improve fatigue and cognitive function in up to 59% of patients with ME/CFS [31]. In addition, compounds in the choline-carnitine pathway were reported to be reduced in patients with ME/CFS [30].

### 2.3. Rat Model Generated under a Stress Environment to Induce Fatigue

In order to identify potential biomarkers and investigate molecular mechanisms underlying the development of ME/CFS, Shao et al. established a rat model generated under a stress environment to induce fatigue as a model of ME/CFS patients [32].

Four methods (restraint-stress, forced exercise, and crowded and noisy environments) were adopted for application in female Sprague-Dawley rats (weight: 200 ± 20 g, 5–6 weeks old) to mimic the multi-factorial pathogenesis of ME/CFS. Rats were exposed to these conditions for 4 weeks. For restraint-stress, rats were fixed individually in a polyvinyl chloride tube (20.0 cm in length, 5.0 cm in diameter) for 4 h. For forced exercise, the rats were forced to run on a treadmill (20 m/min) for 1 h. For a crowded and noisy environment, the 10 rats were housed together in a standard rearing cage, whereas those in the control group were housed in individual cages and were also exposed to rock music for 12 h each day [32]. Urine samples were analyzed by gas chromatography-mass spectrometry (GC-MS). Compared to the control rats, the model rats in the stress environment had significant weight loss as well as significantly altered evaluation indices in the behavioral test, including the time of searching for the platform in the Morris water maze test, number of times and standing events of open-field test, and the motionless time of tail-suspension test, hence indicating chronic stress that resulted in physical and mental (complex) fatigue in the rats [32]. In the model where rats were in a stress environment similar to that of the ME/CFS pathology, α-ketoglutaric acid was markedly more decreased than in the control group. α-ketoglutaric acid is related to the TCA cycle [32], which represents the intersection of catabolism of sugar, fat, and amino acids. The intermediate metabolites of the TCA cycle are also the origins of many biosynthetic pathways. Therefore, the TCA status is a comprehensive reflection of energy metabolism in the body [32] and the model rats in the stress environment showed dysfunction in the TCA cycle metabolism, thereby explaining the fatigue symptoms in ME/CFS. Dysfunction of the TCA cycle has been reported in earlier metabolomic research that used plasma samples from patients with ME/CFS [33].

The major biochemical pathways associated with significant metabolites in this section are summarized in Table 1.

## 3. Metabolomic Analysis in Patients with ME/CFS

In recent years, there has been an increase in studies involving metabolomic analysis for the purpose of developing objective diagnostic biomarkers that reflect the pathophysiological mechanisms of ME/CFS. Metabolomics provides direct small-molecule information and the results can provide immediately actionable treatment information using readily available small-molecular nutrients, cofactors, and lifestyle intervention, which may, in turn, be useful for both diagnosis and personalized treatment [34].

This section aimed to review human metabolomic analysis based on the type of samples used. 

### 3.1. Metabolomic Analysis Using Human Cerebrospinal Fluid Samples

Several magnetic resonance spectroscopy (MRS)-detectable metabolites are fairly well-validated proxies for inflammation, metabolism, and brain health, and are, therefore, of particular potential interest for studying neuroinflammation in ME/CFS. Various studies have used MRS imaging and looked into a range of brain areas and measured a wide variety of metabolites in ME/CFS [35]. Lactate was found to be increased in ventricles in ME/CFS [36,37,38]. Lactate is an end-product of oxidative metabolism and is, therefore, a potentially interesting biomarker for metabolism-associated illness, such as ME/CFS. Lactate levels in healthy brain tissue are too low to be detected by conventional MRS at 1.5T or 3T magnet strength; however, when measured in ventricular cerebrospinal fluid, elevated lactate is associated with neuroinflammation [39,40].

In addition, choline is found to be increased in the left basal ganglia and occipital cortex in ME/CFS [41,42]. Choline is important for the maintenance of membrane health and is a potential marker of BBB status [43]. It is considered a marker for neuroinflammation, owing to its relationship with glial activation and BBB permeability [39]. These findings indicate the strong involvement of inflammation in the pathology of ME/CFS.

### 3.2. Metabolomic Analysis Using Human Urine Samples

The metabolome analyses of urine samples using nuclear magnetic resonance (NMR) spectroscopy reported a glycolytic anomaly in ME/CFS [44]. According to studies that performed metabolomic analysis by NMR using serum samples in addition to other urine samples, reduced glycolysis inferred a reduced utilization of pyruvate and acetyl CoA in the citric acid cycle. The use of amino acids may be through glutamate forming 2-oxoglutarate via aspartate transaminase (AST), which transfers the amino group to oxaloacetate to form and accumulate aspartate. This inefficient energy production together with a lowering of amino acid levels may be important in producing a fatigue phenotype in patients with ME/CFS. The observed increase in creatinine production may provide a means of anaerobic ATP energy for muscles, as well as a mode of nitrogen removal from deaminated amino acids [45]. A previous study reported that an inhibited glycolysis pathway exists in patients with ME/CFS, along with an oxidative stress pathway and a reduced level of amino acids [45].

### 3.3. Metabolomic Analysis Using Human Blood Samples

A broad-spectrum metabolomics study has shown ME/CFS to be a hypometabolic response of high concern to environmental stress that traces to mitochondria [34]. Mitochondrial function is important for energy metabolism [34,46,47,48]. Mitochondria are multifunctional organelles that play a critical role in energy harvesting, transformation, and storage, as well as other intracellular signaling processes [49].

Previous studies used metabolomic analyses to report a decline in the energy metabolism of patients with ME/CFS [30,47,50,51].

The TCA cycle, which in part generates energy from lipids and sugars to make ATP [47] is a pathway that consistently surfaces in ME/CFS metabolomics analysis across platforms and populations. Since fatigue is a major debilitating symptom of this disease, the energy metabolism of such patients has been speculated to be dysfunctional [51].

In addition, taurine and hypotaurine metabolism as the central compound of this pathway, is reported to be dysregulated in ME/CFS [51]. A decrease in the concentration of these metabolites might be reflective of the general effect on the pathway and consequences associated with the general metabolism of the body. Taurine has a number of functions in skeletal muscle, retina, and the central nervous system, and its concentrations may be relevant to the altered amounts of lipid metabolism besides being a part of the primary bile acid biosynthesis pathway, which physically supports the digestion of dietary fat [51].

A study that performed metabolomic analysis to elucidate the functions of the immune system and nervous system in patients with ME/CFS [52] demonstrated that acyl-cholines and steroids were decreased in the patients with ME/CFS compared with levels in healthy subjects. A decrease in long-chain acyl-cholines could explain a disruption in blood pressure regulation, manifested by dizziness, lightheadedness, blurred vision, and near-syncope when assuming and maintaining the upright position. These symptoms are part of those observed in ME/CFS and are presumed to reflect metabodynamics. Furthermore, cortisol, cortisone, and corticosterone were found to be lower in patients with ME/CFS: they have roles in stress response, memory, and inflammation. In fact, there have been reports of abnormal inflammation in ME/CFS cases [35,53] and higher levels of CCL11, CXCL10, IL-7, TNF-α, and TGF-β-1 were significantly associated with higher levels of impaired cognitive processing and musculoskeletal pain [53]. This implies that the results of metabolomic analyses reflect the pathophysiology of ME/CFS.

Kume et al. validated the utility of plasma metabolomics analysis in a rat model of relatively long-lasting fatigue using CE-MS and they found a decrease in energy metabolism with a change in urea cycle metabolism, as well as changes in the levels of amino acids, including BCAAs [28]. Based on these findings, we performed a metabolomic analysis with plasma obtained from patients with ME/CFS and healthy controls using capillary electrophoresis time-of-flight mass spectrometry (CE-TOFMS) [33]. The analysis was divided into study 1 (that used a training data set) to check the metabolites specific to ME/CFS and study 2 (that used a test data set) to check the validity of the results of study 1, and each study involved different subjects. Comparing the results from study 1, between the patients with ME/CFS group and the healthy subject group, there were differences in the concentrations of pyruvate in glycolysis as well as in those of citrate and isocitrate in the first half of the TCA cycle and ornithine and citrulline in the urea cycle, which led to the speculation that patients with ME/CFS undergo metabolic changes specific to the pathology of fatigue during the first half of the TCA cycle and the urea cycle. Next, a support vector machine-feature selection (SVM-FS) analysis, which is a pattern recognition technique, was performed on all metabolites quantified by CE-TOFMS and the results indicated that isocitrate, pyruvate, ornithine, and citrulline were useful for distinguishing patients from healthy individuals. In particular, the increase in pyruvate concentration and decrease in isocitrate concentration, as well as the increase in ornithine concentration and decrease in citrulline concentration suggested that there is (1) a decrease in the flow of metabolites from glycolysis to the TCA cycle and reduced function of the first half of the TCA cycle; and (2) a functional change in the urea cycle, involved in the pathology of fatigue [33]. Therefore, as an indicator of metabolic changes, we established two types of metabolite concentration ratios, namely pyruvate/isocitrate and ornithine/citrulline, and compared these between the patient group and healthy subject group in study 1 and study 2. Both metabolite ratios were significantly higher in the patient group, which suggested that these ratios are useful markers for distinguishing between the two groups. Therefore, we created a multivariate analysis model in order to test the accuracy of ME/CFS diagnosis based on the two types of metabolite ratios and determined the area under the curve (AUC) values by the receiver operating characteristic curve (ROC) analysis. As a result, the AUC value of the indicator that combined the two metabolite ratios was 0.801 (*p* < 0.0001) in study 1, and 0.750 (*p* = 0.0069) in study 2, demonstrating its ability to distinguish between patients and healthy subjects with high precision and its possible contribution as an effective biomarker to objectively diagnose ME/CFS [33].

Fluctuations in the concentration of metabolites in plasma reflect the metabolic changes occurring in each organ. In this original research study, we were able to identify metabolic changes specific to the pathology of fatigue through metabolomic analyses using plasma samples. In other words, the pathology of fatigue is generated due to the functional impairment of the first half of the TCA cycle; this was inferred based on the increase in pyruvate concentration and decrease in isocitrate concentration, which ultimately reduces the ATP levels produced. Therefore, the metabolism of ornithine to citrulline in the urea cycle becomes suppressed and the flow towards glutamine metabolism becomes enhanced as a process to compensate for the decrease in energy production [33]. In addition, intracellular uptake of glutamine is also enhanced and the glutamate levels increase, thus leading to the speculation that there is a mechanism to compensate for energy production in the second half of the TCA cycle, whereby a new metabolic pathway (fatigue metabolism) is created via the γ-aminobutyric acid (GABA)-succinic acid cycle [28]. Along with the metabolomic analysis report of the animal model of combined fatigue [28], we are in the process of confirming the existence of a metabolic pathway considered to be specific to the above-mentioned fatigue pathology. It is also suggested that the enhancement of such a metabolic pathway compensates for decreased energy production while also inducing oxidative stress and inflammation in tissues and bringing about cytotoxicity [28]. Therefore, metabolic changes taking place during fatigue may be thought to be involved in inducing fatigue sensation by increasing inflammatory cytokines in blood and activating microglia in the brain.

Based on the two kinds of metabolite concentration ratios (pyruvate/isocitrate and ornithine/citrulline) reported by Yamano et al., which indices [whether the energy-producing system (glycolysis/TCA cycle) or the urea cycle], have undergone functional impairment can be predicted, and this might lead to the realization that personalized medicine is best suited for the problem(s) of each patient.

The major biochemical pathways associated with significant metabolites in this section are summarized in Table 2.

## 4. Conclusions

Common metabolic fluctuations were observed in fatigued animal models and human patients with ME/CFS and these findings could contribute to the elucidation of the pathophysiology of ME/CFS. Biomarker research, to distinguish between patients with ME/CFS and healthy individuals, is still evolving. In previous studies, reactive oxygen metabolite-derived compounds (d-ROMs) [54] in the blood, exosomes and inclusion proteins/micro RNAs [5], monocyte number, and lipoprotein profiles have been reported to be informative markers for discriminating patients with ME/CFS from healthy controls [55]. Furthermore, inflammation and immune system activation have been suggested by many previous studies to be the root causes of ME/CFS, and the results from many such studies have shown elevation of cytokines and lymphokines in plasma [56]. Using positron emission tomography (PET), neuroinflammation was detected in wide-spread brain regions of patients with ME/CFS, which was associated with the severity of the specific neuro-psychologic symptoms [57].

We believe that in future, it will be possible to establish highly precise objective diagnostic biomarkers for ME/CFS, exhibiting diverse pathologies through the implementation of research that would integrate metabolomic markers reflecting the specific metabolism underlying the pathophysiology of fatigue with highly precise in vivo biomarkers.

## Figures and Tables

**Table 1:** Summary of major biochemical pathways with significant metabolites across fatigued animal models.

Type of Animal Model	Sample Source	Major Biochemical Pathways	References
Rat model of excess fatigue	Plasma	Arginine and proline metabolism Fatty acid transportation and lipid catabolism Phenylalanine metabolism Lecithin metabolism	Zhang et al., 2010 [29]
	Urine	Arginine metabolism Protein catabolism Nicotinate and nicotinamide metabolism Pantothenate and CoA biosynthesis	
Rat model of fatigue	Plasma	Branched-chain amino acid metabolism Urea cycle Proline metabolism TCA cycle	Kume et al., 2015 [28]
Rat model generated under a stress environment to induce fatigue	Urine	TCA cycle Alanine, aspartate, and glutamate metabolism Steroid hormone biosynthesis	Shao et al., 2017 [32]

**Table 2:** Summary of major biochemical pathways with significant metabolites across patients with ME/CFS.

Sample Source	Major Biochemical Pathways	References
Ventricular cerebrospinal fluid	Glycolysis	Murrough et al., 2010 [36] Shungu et al., 2012 [38] Natelson et al., 2017 [37]
Urine	Glycolysis	Armstrong et al., 2015 [45]
Plasma	Tricarboxylic acid cycle Urea cycle	Yamano et al., 2016 [33]
	Amino acid metabolism Taurine metabolism Glyoxylate and dicarboxylate metabolism Pentose phosphate pathway Ascorbate and aldarate metabolism Glycolysis and gluconeogenesis Citrate cycle Starch and sucrose metabolism Galactose metabolism Pyruvate metabolism Purine metabolism Lipid metabolism	Germain et al., 2017 [47]
	Energy metabolism Choline-carnitine pathway Lipid metabolism	Nagy-Szakal et al., 2018 [30]
	Amino acid metabolism Tricarboxylic acid cycle Pyrimidine metabolism Purine metabolism	Germain et al., 2018 [51]
	Amino acid metabolism Lipid metabolism Xenobiotics	Germain et al., 2020 [52]
Serum	Amino acid metabolism	Fluge et al., 2016 [50]
